# Odontological Society of Pennsylvania

**Published:** 1895-09

**Authors:** 


### ODONTOLOGICAL SOCIETY OF PENNSYLVANIA.

A regular meeting of the Society was held March 9, 1895, President Peirce in the chair.

Owing to the late arrival of the essayists, the president called on the members present for incidents of practice. In response Dr. Jeffries said,—

I would like to speak of a gentleman who had suffered for about eighteen months with neuralgia. He had been to his dentist a number of times and had his teeth examined to find out, if possible, the cause. In this emergency the upper wisdom-tooth on the painful side had been taken out. That was the history when he came to me to see if I might give him relief. There were no cavities or dead teeth on the affected side, but a broken root of the extracted wisdom-tooth remained, which I removed.

In sounding the bottom of the old socket I detected the crown of a tooth opposite the posterior buccal root of the second molar. I endeavored for half an hour to extract it without losing the second molar. Finding this impossible, I took out the second molar, and then without difficulty extracted a second wisdom-tooth that was lying on its side with the crown surface pressing against the roots of the second molar. The gentleman was cured of his neuralgia.

Dr. Peirce, the president, then related the following: A somewhat analogous case was that of a lady of about forty years of age, who had been wearing an artificial denture for eight years. A tumor appeared, which grew to some size, and the patient suffered considerable neuralgic pain. I made an incision in the tumor, and took out three teeth impacted together. One was nearly normal size, the second much smaller, and the third was very small. Relief was afforded after these were taken out.

They were located on the right side of the upper jaw, and a hole had been cut in the plate so as to give room to the tumor.

The larger one, which alone could be classified, appeared to be a third molar; the other two were much smaller and could not be designated as any particular tooth. The operation gave entire relief.

Dr. B. Holly Smith read a paper entitled "A Plea for Moderation."

(For Dr. Smith's paper, see page 547.)

DISCUSSION.

*Dr. Henry Leffmann.*—I am not prepared to differ very seriously with the paper, but I am not entirely in accordance with some of its statements; I mean as to the value of a college education, what we may call, for want of a better term, classical training. The value of this training in its bearing upon professional work is a very difficult thing to determine, and we have very incomplete data upon which to form a judgment.

It is undoubtedly true that genius asserts itself in a variety of ways, and that very able men have passed into the profession without much preliminary training, but I think we must look at preliminary training in its selective effect. Some years ago Dr. Pepper, in a paper on medical education, made a very wise remark. He said that a preliminary examination might be of value in deterring unworthy students from entering the college, as the mere mention of requirements might be sufficient to keep out certain incompetent persons. Appearing before the community as a profession, it is important that the merely practical phase ought not to be given too large a place. Dentists want to be more than simple repairers of teeth and manufacturers of artificial dentures; they want to carry a degree which is a degree of honor; they want to take rank as educated men, and so the colleges ought to insist upon some preliminary requirement other than those merely of the lowest school grade. Of course, the colleges do not pretend to take men who are not familiar with reading, writing, arithmetic, and some fair knowledge of the phenomena of life; but I recollect in my early days, when I was acting as quiz master, having a gentleman in my class who did not know the nature of a decimal fraction.

It seems to me that we might at least insist on some kind of a high-school education so that our students might be capable of understanding the more difficult departments of science which are laid before them. There is in the minds of many persons the thought that dentistry may be before long a specialty of medicine, and that a medical degree will be required as part of the dental degree. If so, we shall certainly have to prepare the profession for that expansion.

There is one thought that struck me, which is in reference to the question of the poverty of students. I think our charity at times is a little too wide in educational matters. When it comes to providing men with means for making their living, I question whether the State and college is not going too far in encouraging those who are simply unable to pay their way. It may be true, in rare cases, that some valuable minds may be lost to the profession, but in such cases I think the qualities will be such as to attract private assistance. A part of a good education is the money it costs, and my experience, which extends over twenty or twenty-five years of work in several collegiate institutions, leads me to believe that the assistance which is given in colleges to fit the student for professional life is often badly placed. Very often it brings into a profession some one who is better fitted for a humbler calling.

As to the extension of the dental course to four years, I myself am not in favor of it, but I think that what we want to do is to raise the preliminary requirements very decidedly; have some system by which we may gather together in the lecture-room a class of students who are thoroughly capable of appreciating the work that goes on before them. It is a very easy thing to say that a man may be a good dentist and yet not able to read Latin, but at the same time, in the course of his study of Latin and other collegiate branches, his mind becomes developed so that he makes a better dentist. I once said to a chemistry class, in explaining a difficult problem, that probably some great medical man (I gave his name) did not understand the subject upon which I was lecturing and was no worse practitioner for it, but that at the period of his student life at which he would have been expected to know the abstruse point he would have known it. While in after years a person may not be able to construe a sentence of Latin, yet in his work as a practitioner he will be better developed and better able to do the work of the profession if he has construed such sentences in his student days.

*Dr. Bonwill.*—If I had been obliged to understand Latin and Greek I would never have been a dentist to-day, although it is a splendid thing to know them in order to understand the derivation of words if for nothing else, but a man must have mechanical genius.

There should be no extension in the period of tuition; with a man of genius it becomes unbearable. Only by getting into actual work and coming in contact with older practitioners will he be fully equipped to do his work. Further, there should be no such thing as a post-graduate course, as the colleges are supposed to have the best teachers procurable, and in setting up a post-graduate course it is a reflection on the present institutions of learning.

*Dr. Smith.*—I thank the gentlemen for the courteous treatment of the paper. I was well aware before the paper was presented that it would meet with opposition. I know that the gentleman who discussed the paper at some length has the popular side of the question, and I do not desire to take any further issue with him than is taken in the paper. My claim and my position was that we may rest upon our oars and that we do not force in any immoderate way the good movement already started. I do not think any gentleman in the room will charge me with desiring too low a standard in the dental profession. On the contrary, all I have ever said or done has been in the line of progress. But there are many incongruous and inharmonious plans and suggestions offered as to the education of dental students, and it is with the view that some harmonious conclusion may be gotten from them that this paper is presented to you.

Dr. William Shaw Twilley, of Baltimore, Md., next read a paper entitled, "The Census of 1890 and the Dentists."

(For Dr. Twilley's paper, see page 551.)

DISCUSSION.

*Dr. Richard Grady.*—As Dr. Twilley has said, one of the census enumerators waited on me in August, 1890, and on the 1st of September, 1890, I published in one of the Baltimore papers an article entitled "Are Dentists Manufacturers?" I took the ground that everything which the dentist makes in his practice is surgical; that a filling is surgical. This matter had been gone over many years ago in the census of 1880. The head of the census department at that time was General Walker, who is now and was then president of the School of Technology, Boston. He was also editor of the report of your International Exhibition of 1876, and chief of the bureau of awards. In this very book the work of dentists was classed as purely surgical, and it was so classed not by dentists but by surgeons.

I represented in 1890 that the dental surgeon cannot properly be subjected to annoyance and investigation in respect to his professional work any more than the surgeon in other specialties of the medical art; that he stands in delicate and confidential relations to his patients; that the demand for reports from dental practitioners was novel and unjustifiable; that the superintendent of the census of 1880, after correspondence, determined to omit "dentistry in all its branches" from tabulation, and caused all schedules on file to be endorsed to that effect and returned to their respective makers.

The word *surgery* denotes handwork in connection with the art of healing, whether manual or instrumental. I stated that so early as 1876 the surgeons classified the following specimens as purely surgical: "Pivot teeth, dentures, complete and incomplete, with and without spiral springs, the various methods of retention by atmospheric pressure and by clasps, and a series of orthopædic apparatus for the correction of congenital malformations of the palate, loss by scrofula, and syphilitic necrosis."

The appliances made in the operating-rooms and laboratories of dentists, which the census office of 1890 claims as "mechanical," are made only after examination of the patient, or casts of the parts for which the appliance is needed. In some cases, teeth or soft tissue require removal in addition. When the appliance is finished it is useless and of no value except to or for that particular patient. These appliances are thus surgical. They are the result of diagnosis, prognosis, and handwork.

No schedule was left with physicians and surgeons. Why, then, should dental practitioners be called upon to give information in regard to the personal treatment of their patients?

In reply to these questions the census office said, writing to its agent at Baltimore, September 18, 1890, "In reference to mechanical dentists, you are informed that the term is considered by this office to embrace all branches of the profession except the extraction of teeth, and should be so reported on General Schedule No. 3."

I took exception to that definition. Time went on, and the census office, in October, 1890, wrote to your Philadelphia County Dental Society giving another definition of *mechanical dentistry*,—namely, "That branch of the profession which includes the manufacture of artificial dentures, such as crowns, plates, and caps." The census office was also in error in claiming that at the census of 1880 opposition was uniformly withdrawn when proper explanations were made to the persons averse to giving the information required.

The facts are that the superintendent of the census of 1880, in deference to a general protest, determined to omit from the tabulations dentistry in all its branches.

On December 30 the census office decided to order the census officials to demand answers to all inquiries, and to prosecute those who should refuse to comply. Mr. Williams, a special agent of the census office, said to a *Sun* representative that the same questions that the Baltimore dentists objected to were being put to the dentists in other cities, and willing replies were being received every day. Determined not to let such an official statement go unchallenged, I wrote, "The iteration and reiteration in Washington that the dentists of Baltimore alone resist the purpose of the census-office inquiry is untrue, as the Philadelphia County Dental Association has unanimously adopted resolutions instructing its members not to attempt the impossibility of making any returns, and has presented its protest at Washington."

Now, I would like to show you gentlemen how correct you were in taking that stand. I don't know whether you are familiar with the Extra Census Bulletin or not; I never saw it until last night. In this bulletin this acknowledgment is made: "A large number of reports were received from dentists which purported to represent only the mechanical work, but it is evident that in many cases operative dentistry had been included. This fact, combined with the strenuous objection on the part of the profession to give any information whatever of the character required by the census schedule, on the ground that the census law does not seek to secure the returns for professional services, caused the office to discontinue its efforts to obtain further reports for the mechanical branch of the profession. The statistics published are those which were secured prior to the objection referred to."

I grieve to say that just when this news of hope afforded unparalleled opportunity for success, the public press, January 14, 1891, announced from Washington that "Baltimore dentists answer willingly," and that Mr. Williams, special agent of the census office, "believes the entire difficulty is ended and that the dentists of Baltimore no longer hesitate about furnishing the desired information." This was partially true. Nothing could have been more startling than the sudden admission of a committee of some Baltimore dentists that it was for the good of all concerned that practitioners should furnish the information which the census office desired. A feeling of depression, if not of indifference, followed the division of sentiment in Baltimore.

I answered this publication as follows: "In reference to the paper signed last week in Washington, the three dentists who signed the paper at the census office did not voice the sentiments of the incorporated bodies of the city and State, the Association of Dental Surgeons of Baltimore City, and the Maryland State Dental Association, which have unanimously adopted resolutions declining to make returns, which resolutions have never been rescinded, and that the president of the Philadelphia Dental Society has written under date of January 16, that 'the society has no intention of receding from the position taken.' In no case that I am aware of has any member of the Society filled out a blank of the manufacturers census since the passage of the resolution."

In three weeks after my communication the error was atoned for, since Lawyer Venable gave as his opinion "that there was no penalty for refusing to answer the questions, and the dentists might either ignore the whole matter or answer generally that they did not operate manufacturing establishments."

So matters rested until the Willcox Bill was introduced in March, 1892, when I wrote the following, which was unanimously adopted by the Maryland State Dental Association, May, 1892:

WHEREAS, a bill has been introduced in Congress, which provides that any person who shall neglect or refuse to give information to census officers shall be subject to a fine not exceeding ten thousand dollars, to which may be added imprisonment for a period not exceeding one year; and whereas, the public press reports that the bill referred to "will be used in the nature of thumbscrews and spiked boots to enforce replies," and further, that "dentists of Baltimore who by their refusal to answer certain questions will be the first victims of the inquisitorial machine if the bill is passed," therefore,

*Resolved*, that this Association reaffirms the resolutions unanimously passed November, 1890, protesting against the action of the census bureau in classifying dentists as manufacturers.

*Resolved*, further, that the chairmen of the several standing committees as now constituted, be appointed to convey the sentiments of this Association to the Senators and Representatives of Maryland in Congress, and urge them to have the Willcox Bill amended, so as to omit dentistry in all its branches from the tabulations of the census.

*Dr. Thomas.*—History wants to be correct in all its bearings, and the history of the efforts made by that committee is not correct as it stands to-day, so I am very glad that Dr. Twilley, of the committee appointed by the American Dental Association, is making an effort to establish facts upon the correct basis.

As it stands to-day, any one who reads the report would think that 1892 was the beginning of the effort to establish the standing of the dental profession, and that Dr. John B. Rich was the man who did it; but as Dr. Twilley says, two years before, in 1890, the effort was made by the Baltimore men, Drs. Twilley and Grady, and others, seconded by Drs. Boice and Faught of this city. They did not succeed, however, in arousing sufficient interest in the dental profession, and the proper stand was not made. Later on committees were appointed by various societies over the country who should go to Washington and attempt to defeat the Willcox Bill. I happened to be the representative with Drs. Faught and Deane selected to represent this Society.

In my address before the Senate committee, I said that the dentist, as an individual, did not by any means consider himself better than a manufacturer, but that the law required more of a dentist than of a manufacturer. The dentist was compelled by law to be an educated man and to have a diploma from a reputable college, while a manufacturer need not be able to write his name. Therefore, in the eyes of the law, the dental profession was on a higher plane than the business of manufacturing. But that was not the point of our argument. The fact was that dentists really manufacture nothing. Everything that comes to their office is already manufactured,—teeth, plates, screws, etc.,—and they simply take them and apply them to each individual case, and the denture has no possible value except to the individual mouth for which it is made. That point carried the day. Senator Eugene Hale said, "You might as well call a physician or surgeon a manufacturer because he fits a splint to a broken limb."

*Dr. Faught.*—Dr. Thomas has referred in his remarks to the fact that this history has never been correct, and it is very essential for the future of the profession that this history be intelligently written and accurately stated. We are all aware that the American Dental Association has appointed a committee to take steps to do this, and therefore it is essential now, before the meeting at Asbury Park next summer, that all the facts be brought out in the local societies.

One thing I want to say by way of correction to Dr. Thomas's statement, that the efforts made prior to 1892 had not carried the profession with it. That is not exactly the case. There were not two separate efforts, one beginning with my work in 1890 and extending up to 1892, and another taking inception in 1892 under the direction of Dr. Thomas. The work which was done by me, largely as an officer of the Philadelphia County Dental Society and partly as a member of the Odontological Society from 1890 to 1892, certainly was not a popular movement, but it required courage on the part of the few, and owing to their steadfastness and aggressiveness the foundation was laid for the successful culmination under Dr. Thomas in 1892; but I deemed it unnecessary to go to Washington, as my ideas were already before the Census Bureau in writing, while Dr. Thomas, too, being there (for the first time), could command attention with new facts.

On October 20, 1890, I introduced the first set of resolutions opposing the census classification to the Philadelphia County Dental Society, and at that time it met with bitter opposition. I remember how Dr. Boice said we were going into a pretty big fight; that we would have a great deal of trouble and take a good deal of time and expense to successfully contest the matter through the courts, and we would probably be mistaken if we expected to have the support of the profession. I insisted, however, and Dr. Boice withdrew his opposition and voted for the resolutions. The resolutions were adopted, and then on November 1 I came to the Odontological Society and introduced similar resolutions under threats of the Census Bureau. Those resolutions were afterwards rescinded by this Society, in so far as they controlled the individual action of the members. I was absent at the time. At the next meeting I again insisted, and the resolutions were again put on the minutes. In the mean time the census agent told me the resolutions had been rescinded, and I told him to go look at the minutes and he would find they were still there on the statute book, and he so found them. Then it was I went to Baltimore May 2, 1891, and afterwards up to Albany. Then the movement had become popular throughout the country, and you appointed the Thomas committee, which brought to a satisfactory conclusion the effort which the few had so pluckily striven for so long.

I do not want it to go on record and be understood that all the work which led up to it for two years was for nothing, and that the result was obtained without this battle. There was bitter opposition for two years, and it was only by the persistent action of myself and the few others that the matter was kept agitated and the result finally obtained at Washington in 1892.

